# Evaluation of Er:YAG, Er,Cr:YSGG and diode lasers irradiation on radicular dentine fatigue strength using modified endodontic laser tips

**DOI:** 10.2340/biid.v12.44960

**Published:** 2025-12-17

**Authors:** Jaana Hannele Sippus, Marcelo Capitanio, Mustafa Murat Mutluay, Rene Franzen, Arzu Tezvergil-Mutluay

**Affiliations:** a Department of Cariology and Restorative Dentistry, Institute of Dentistry, University of Turku, Turku, Finland; b Biomaterials, and Medical Device Research Program, Adhesive Dentistry Research Group, Biocity, Turku, Finland; c Finnish Doctoral Program in Oral Sciences (FINDOS), Institute of Dentistry, University of Turku, Turku, Finland; d Department of Oral and Maxillofacial Diseases, Faculty of Medicine, University of Helsinki, Helsinki, Finland; e AALZ Aachen Dental Laser Center, Aachen, Germany; f Dental Department, Medical Faculty, SFU Sigmund Freud University, Vienna, Austria; g Oral and Maxillofacial clinic, Turku University Hospital, TYKS, University of Turku, Turku, Finland

**Keywords:** dentine, fatigue, lasers, endodontics

## Abstract

Laser-assisted endodontic treatments have gained popularity over the last decade. This study evaluated the flexural strength (FS), fatigue resistance, and surface characteristics of root dentine after laser-assisted endodontic protocols.

Forty extracted, caries-free canines were used to prepare root dentine beams (*n* = 37/group). Beams were irradiated with (1) Er:YAG 2,940 nm (20 mJ, 0.3W, 15 Hz, 50 ms), (2) Er,Cr:YSGG 2,780 nm (2.25W, 50 Hz, 140 ms), and (3) 940 nm diode laser (1W, CW). The non-irradiated beams served as control group. Both erbium groups were treated with laser-activated irrigation with radial firing tips (RFTs); meanwhile, the diode group irradiation protocol, using RFT, was dry. Specimens underwent quasi-static loading (*n* = 12) and cyclic loading for fatigue behaviour (*n* = 25) using 4-point flexure test. Scanning electron microscopy analysis was performed. Data were analysed using analysis of variance and Kruskal-Wallis tests (α = 0.05).

No significant difference was found in FS or fatigue resistance after laser-assisted treatment (*p* > 0.05), but endurance limits improved by 18% (Er:YAG) and 19% (Er,Cr:YSGG) compared to controls.

These findings suggest that Er:YAG, Er,Cr:YSGG, and 940 nm diode lasers, when applied with recommended parameters, do not compromise dentine fatigue strength. Therefore, they may be safely integrated into root canal treatment protocols.

## Introduction

The success of endodontic therapy depends on the complete removal of contaminated organic and inorganic tissues from root canals and elimination of bacterial contamination [1]. Chemical irrigants such as sodium hypochlorite (NaOCl), chlorhexidine (CHX), ethylenediaminetetraacetic acid (EDTA), and MTAD (mixture of doxycycline, citric acid, and a detergent) are commonly used in conjunction with mechanical instrumentation to achieve adequate cleaning. Although the combination of NaOCl and EDTA is considered the traditional technique for endodontic debridement [2], studies have shown that chemical-mechanical preparation is often insufficient, particularly in the apical third of the root canal [3].

Recent advancements in laser technology have introduced new possibilities in endodontic treatments. Diverse lasers have been examined for their capability to improve root canal cleaning and disinfection, supplementing conservative chemo-mechanical procedures [4–7]. Meanwhile, different laser wavelengths present unique optical properties, and enhancing their application is essential. While some lasers demonstrate strong bactericidal effects, others have been suggested as a complementary method in enhancing the removal of smear layer and necrotic tissue debris [8, 9].

High-intensity lasers, such as erbium lasers (Er:YAG 2,94 mm and Er,Cr:YSGG 2,78 mm) have gained popularity for their ability to eliminate microorganisms and enhance dentinal permeability by removing the smear layer [5, 6, 10, 11]. In laser-activated irrigation (LAI), laser irradiation, using low energy with short pulses, generates micro-cavitation bubbles in the irrigant, leading to a high-speed fluid motion and improved cleaning efficacy. These lasers are highly absorbed in water and hydroxyapatite, enabling efficient hard tissue ablation with minimal thermal damage, when used with appropriate parameters and water spray [12–15]. Er:YAG lasers have a water absorption coefficient approximately 300% higher than Er,Cr:YSGG lasers, resulting in more rapid energy dissipation in a thin water layer and shallower tissue penetration of only a few microns. Despite this, Er,Cr:YSGG lasers may achieve greater mass ablation per pulse due to the high absorption in water and hydroxyapatite. However, they can also cause temperature rises during ablation, particularly when the irrigant reaches boiling point, potentially leading to thermal damage. The resulting pressure waves contribute to smear layer removal [15].

Since 1999, erbium lasers have been recognised as effective adjuvants for debris and smear layer removal, improving dentine permeability and enhancing cleaning efficiency [16]. Recent studies underscore the role of water in facilitating smear layer removal [5, 17–19] and confirm the superior cleaning efficiency of erbium lasers compared to EDTA irrigation, hand activation, or ultrasonic-activated irrigation [15, 20–23].

In addition to erbium lasers, the 940 nm diode laser is widely used for bacterial decontamination, often parallel with Er,Cr:YSGG and in dual-wavelength protocol (940 + 2,780 nm in same fibre) [3, 24, 25]. This 940 nm wavelength is highly absorbed by melanin, haemoglobin and oxyhaemoglobin, and features a distinct absorption peak in water, compared to other diodes (810–980 nm). Diode lasers penetrate deeper in water-based tissues (1–3 mm), depending on wavelength and tissue type. Their interaction with bacteria involves thermal effects and targeting of pigmented microflora [26–29]. Unlike erbium laser, diode lasers do not produce cavitation and rely on chemical irrigants for smear layer removal. Additionally, diode lasers do not enhance adhesion to endodontic sealers or reduce apical leakage [30, 31]. Nevertheless, diode lasers are considered safe, with minimal temperature rise during irradiation [30]. When applied using circular movements from the apical to coronal regions, and with adequate cooling intervals, they facilitate safe and effective bacterial decontamination [3, 6, 24, 32, 33].

Early endodontic laser tips, such as bare-ended fibre tips directed the laser energy straight towards the apex, resulting in an uneven energy distribution and potential damage beyond the apical area [34, 35]. The advent of radial firing tips (RFT) for Er,Cr:YSGG lasers (Waterlase, Biolase Inc, Foothill Ranch, CA, USA) marked a significant improvement. These conically shaped tips emit energy in a 360° pattern, enabling uniform interaction with root canal walls and enhancing biofilm removal while maintaining safety [20, 21, 24, 36]. RFTs also reduce temperature rise and improve dentine cleaning without compromising structural integrity [21].

Given that cavity preparation often leads to cracks and structural damage, the mechanical behaviour of dentine after endodontic treatment is critical [37–40]. Prior studies have assessed dentine fatigue degradation [37] and compared fracture forces between dentine treated with high-speed drills and erbium lasers [40]. However, these findings were not as extreme as in previous results [55], due to the testing setup.

The aim of this study was to evaluate the flexural strength (FS) and fatigue resistance of root dentine following laser-assisted endodontic protocols. Additionally, the effects of laser application on the dentine surface morphology were investigated. The tested null hypotheses were as follows: (1) laser irradiation using Er:YAG, Er,Cr:YSGG, and 940 nm diode protocols does not affect the 4-point FS of root dentine, and (2) laser irradiation does not affect fatigue resistance of root dentine, and (3) the laser irradiation does not alter the surface morphology of dentine.

## Materials and methods

### Sample preparation

Forty extracted caries-free human canines were stored at 4°C in 0.9% NaCl containing 0.02% sodium azide (NaN_3_) to prevent microbial growth and used within 3 months after extraction. The extracted teeth, obtained from anonymous donors, were exempt from ethical notification according to Finnish law (Tissue Act, section 20). Decoronation was performed, and 148 dentine beams (0.9 × 0.9 × 12 mm) were prepared from the roots using a low-speed saw (Isomet, Buehler, Lake Bluff, IL, USA) under water cooling. For quasi-static setup, sample size was estimated using an online calculator for continuous outcomes (sealedenvelope.com), based on FS data reported in a previous study [41] comparing sound deep and superficial dentine (119 ± 31 MPa vs. 169 ± 28 MPa), with 95% power and a 1% significance level. The sample size for the fatigue test was defined based on previous in vitro studies assessing fatigue strength, which employed similar experimental conditions [42]. The preparation procedure for cutting beams was modified from the description in a study by Staninec et al. [43]. Beam pulpal surfaces were slightly hand-wet-polished with 320-grit SiC grinding paper (Carbimet & Microcut, Buehler Ltd) for 10s for smear layer standardisation. Specimens were re-inspected for possible flaws due to sample preparation using a stereomicroscope (Leica M60) at 40 × magnification and stored in distilled water at 37°C prior to further testing. Samples with detectable flaws were discarded and replaced.

### Laser-assisted protocols

**Figure 1:**
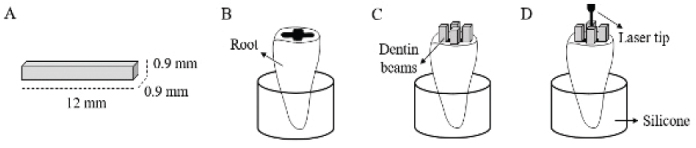
Protocol configuration. (A) root dentine beam preparation; (B) mould imitating the root canal environment; (C) dentine beams positioned inside the root; (D) laser irradiation on the pulpal side of the beams.

Root dentine beams were randomly allocated to four groups (*n* = 37/group) based on the laser type, mode of operation, and laser tip used (Table 1). A custom-made mould was created by embedding a single root within a silicone matrix, with designated space for four beams to mimic clinical conditions (Figure 1). Beams from four different teeth were irradiated simultaneously on their pulpal side while immersed in 0.9% NaCl solution inside the root, following specific manufacturer’s protocols for each laser system and respective tips. The diode group was treated dry. It is important to note that pulse energies were adjusted to account for the varying tip diameters of different laser systems, ensuring a comparable effect across all groups. Experimental groups included beams treated with three different lasers: 940 nm diode, Er,Cr:YSGG, and Er:YAG.

**Table 1:** Experimental groups and operation modes for different laser tips.

Group	Wavelength	Irrigation	Tip	Mode of operation	Manufacturer
Control	None	NaCl with syringe	0.3 mm	Helical motion starting close to apical part 4 × 10s with 10s intervals tot. 4 × 20s. cycles	Biolase, San Clemente, USA
Diode	940 nm	NaCl with syringe in between irradiations	Endo 0.2 mm surgical HP	1W, CW, 0.23 J/cm^2^ Helical motion starting close to apical part 4 × 10s with 10s intervals tot. 4 × 20s. cycles	Epic X, Biolase, San Clemente, USA
Er,Cr:YSGG	2,780 nm		RFT3 0.3 mm Gold HP	2.25W, 50 Hz H, Air 35 Water 25; 0.52 J/cm Helical motion starting close to apical part 4 × 10s with 10s intervals, tot. 4 × 20s. cycles	Waterlase, Biolase, San Clemente, USA
Er:YAG	2,940 nm	NaCl with syringe whole time	PIPS 0.4 mm HP14	20 mJ, 15HZ 0.3W 50 μs (SSP) 4 × 20s; 0.07 J/cm^2^ Static tip inside pulp chamber close to the canal orifices	Lightwalker, Fotona, Ljubljana, Slovenia

The beams in four different groups were treated according to the protocols, as follows:

The Er:YAG 2,940 nm group (Lightwalker, Fotona, Ljubljana, Slovenia) was irradiated applying 20 mJ, 15 Hz, (0.3W), with a pulse duration of 50 μs (SSP) and laser spray ‘off’. A quartz tip with design of 0.4 mm in diameter and 12 mm long, PIPS tip, was utilised. The tip was positioned in the pulp chamber close to canal orifices of the NaCl-soaked root canal and kept still. Throughout the cycles of laser irradiation (4 × 20s), the beams were constantly irrigated with 2 mL of liquid to uphold hydration by a hand syringe with a 25-gauge needle situated above the laser tip in the coronal side of the canal opening, according to the above protocol. For the photon-induced-photoacoustic streaming, it is important that the canal is wet all the time.

Er,Cr:YSGG 2,780 nm laser group (Waterlase, Biolase, San Clemente, USA) was irradiated with settings of 2.25W, 50 Hz H mode (140 ms) and pulse Air 35% + Water 25% using a radial emitting tip RFT3 with a diameter of 0.3 mm. The tip was placed up to the working length, and irradiation was completed at a speed of 2 mm/s till the upper part of the mould. The irradiation process was repeated four times 10s with equal intervals between each irradiation, totally 4 × 20s cycles.

The 940-nm diode laser group (Epic-X, Biolase, San Clemente, USA) was operated at 1W, continuous wave (CW), using an endo tip with a diameter of 0.2 mm applying the same protocol as Er,Cr:YSGG group described above in dry canal.

The control group was irrigated with 5 mL saline solution using a 25-gauge needle. A laser fibre tip with 0.3 mm was inserted in the middle of the mould in working length and moved in helical motion upwards four times with intervals and rinsed with 5 mL saline solution in between and after all four ‘cycles’. This was done without laser irradiation but to mimic the occasional probability of the fibre tip to touch the beam surface during the helical motion and the possible impact on fatigue behaviour during the fibre tip movement even without any irradiation.

During all the treatments, the beams were held in hand and kept still to avoid any movement due to the cavitation effect of LAI.

The difference between the protocols is that Er:YAG with PIPS is applied only in the pulp chamber, while Er;Cr:YSGG and diode tips are inserted in the canal. The laser irradiation is absorbed in the irrigant solution, NaCl in both erbium groups, whereas the diode protocol is dry.

The control group consisted of non-irradiated beams, which were only irrigated with 0.9% NaCl solution. EDTA is a commonly used chelating agent for eradicating the smear layer in endodontics. However, the present study did not use canal irrigation solutions that are capable of removing the smear layer. The aim was to isolate the impact of laser-assisted endodontic treatment on the dentinal surface, as the focus was solely on the effect of laser irradiation on fatigue behaviour. While NaCl was the only irrigant for erbium laser groups and control, the 940 nm diode group was rinsed with NaCl after each laser irradiation. Treatments were according to the manufacturer’s protocols for respective devices. The buccal surface of each dentine beam was marked to indicate the irradiated side for SEM orientation.

### Characterisation of the fatigue behaviour

**Figure 2:**
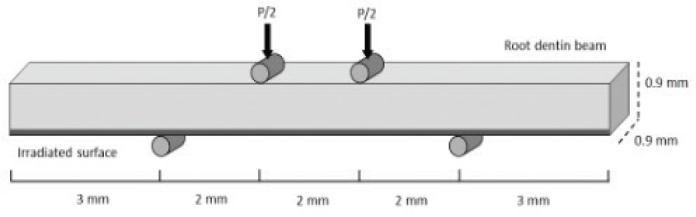
Schematic diagram of specimen configuration and loading arrangements used for characterising the 4-point-flexure at quasi-static loads and stress-life fatigue behaviour at 4Hz at cyclic loads.

A universal testing system (ElectroPlus 1100 Instron, UK) with a load capacity of 250 N and sensitivity of 0.025% was used to perform quasi-static and cyclic four-point flexure to evaluate root dentine specimens. A schematic diagram with the jig, specimen configuration, and loading arrangements is shown in Figure 2. Quasi-static loading was operated at a rate of 0.05 mm/min. Conventional beam theory was used to calculate the FS of the beams expressing the highest measured load (P) in N and beam geometry (width b, thickness h in mm) according to FS = 3Pl/bh^2^, where l stands for the distance from interior and exterior supports (l = 2 mm). Each of the four evaluated groups consisted of 12 specimens (*n* = 12). The same flexure configuration was used with cyclic loading of the specimens under load control using 4 Hz with a stress ratio (*R* = ratio cyclic load from minimum to maximum) of 0.1. The setup for cyclic loading utilised the staircase fatigue method, initiating at approximately 95% of the FS, recognised from the quasi-static loading, following sequential 10% decreases until failure. The process was prolonged until a flexure stress amplitude (MPa), at which the specimens did not fracture within 1 × 10^6^ cycles, was reached. The amplitude for cyclic stress was plotted based on the number of cycles to failure in a log-based format. The data was fitted through a non-linear regression with a Basquin-type model, according to equation σ = A(N) B, where A is the fatigue-life and B is the fatigue-life coefficient exponent. The evident endurance limit was estimated from the models for a fatigue limit outlined at 1 × 10^7^ cycles. For each group, 25 specimens were evaluated.

### Scanning electron microscopy

Samples (*n* = 10/group) that withstood a minimum of 1 × 10^4^ loading cycles were evaluated using scanning electron microscopy (SEM) to characterise the surface morphology and identify the failure. The samples underwent an ultrasonic cleaning process in distilled water for 60 seconds, followed by dehydration through an ascending series of ethanol solutions, ending with 100% ethanol. A final chemical drying process using hexamethyldisilane (HMDS) was performed overnight. Since formalin fixation significantly impacts the enzymatic activity within the tissue, alternative fixatives were used in this study to preserve both tissue morphology and enzyme activity. Subsequently, samples were sputter-coated with gold/palladium and analysed at 10 kV (Phenom ProX, Phenom World). SEM micrographs (3,000 × magnification) were taken sequentially on the tensile side of the beams.

### Statistical analyses

The Shapiro-Wilk and Brown-Forsythe tests confirmed the normality and equality of variance of the 4-point FS and cross- sectional data set. Flexure strengths achieved after quasi-static loading measurements and cross-sectional areas were analysed using a one-way analysis of variance (ANOVA). Fatigue life distribution data were analysed using the Kruskal-Wallis one-way ANOVA on ranks. Significance levels were set at 5% (α = 0.05). Statistical analyses were performed using Sigma Plot version 15 for Windows.

## Results

### Quasi-static 4-point FS

**Figure 3:**
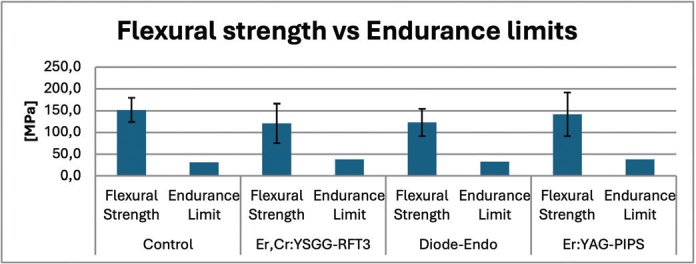
Flexural strength and endurance limits for control and experimentral groups.

A summary of strength outcomes under quasi-static loading is presented in Figure 3. FS values ranged from 121MPa to 151MPa for Er,Cr:YSGG, and control groups, respectively. One-way ANOVA revealed that the laser application protocols had no significant effect on FS (*p* > 0.05).

### Fatigue behaviour and endurance limits

**Figure 4:**
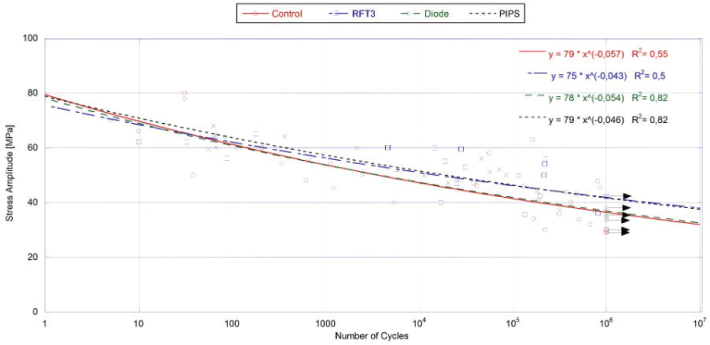
Stress-life diagrams for root dentine specimens after laser-assisted treatments. Note that data points with arrows represent those specimens that reached 1 × 106 cycles, and the test was discontinued. The R2 values represent the coefficient of determination.

Fatigue life data (S-N curves) are shown in Figure 4. Regression analyses using Basquin-type power law models were applied to describe fatigue strength trends. The Kruskal-Wallis test showed no significant differences in fatigue responses between groups (*p* > 0.05). However, Er:YAG and Er,Cr:YSGG groups exhibited the highest fatigue strengths, while the diode and control groups showed lower values. Apparent endurance limits at 10^7^ cycles calculated from stress life constants were 18 and 19% higher for Er:YAG and Er,Cr:YSGG groups, respectively, compared to control.

### Scanning electron microscopy

**Figure 5:**
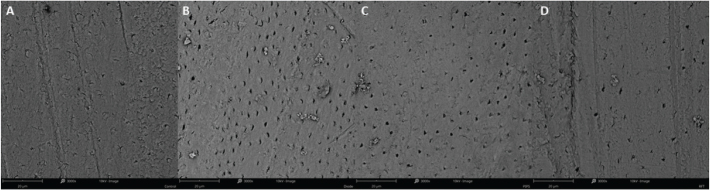
Representative SEM images highlight surface morphological differences between groups. A - The control group displayed a sense smear layer with few visible dentinal tubules. B - The diode laser group exhibited partially open tubules with less smear layer. In contrast, C - the Er:YAG and D - Er,Cr:YSGG groups showed more smear coverage, where a minor part of tubule openings was observed.

Representative SEM images (Figure 5) highlight surface morphological alterations between groups. The control group displayed a dense smear layer with few visible dentinal tubules. The diode laser group exhibited partially open tubules with less smear layer. In contrast, the Er:YAG and Er,Cr:YSGG groups showed more smear coverage, with a minor portion of the tubule openings visible.

## Discussion

This study demonstrated that laser-assisted endodontic protocols using Er:YAG, Er,Cr:YSGG, and 940 nm diode lasers did not significantly alter FS of root dentine. Therefore, the first null hypothesis was accepted. Under quasi-static loading to failure, all laser-treated groups showed comparable fracture strength compared to control, suggesting that none of the laser treatments induced significant defects compromising the structural integrity of dentine. This aligns with the findings from previous research indicating that typical laser fluences used in endodontics remain below the ablation threshold (e.g. 4 J/cm^2^ for Er:YAG), minimising the risk of mechanical or thermal damage. A recent study that employed fluences above the ablation threshold to simulate a cavity floor preparation also confirmed that laser irradiation did not compromise dentinal beam integrity [12]. Although Staninec et al. reported mechanical and thermal damage from a 0.5 ms ultra-short pulse duration in dry conditions with Q-switched Er,Cr:YSGG lasers, compared to pulsed and Q-switched Er:YAG using 35 and 135 ms pulses, these conditions did not reflect clinical practice [43]. In contrast, our study employed clinically relevant parameters, including water cooling and a standard pulse duration of 50 ms for Er:YAG, which are less likely to cause damage. In laser therapy, the mode of operation, including wavelength, fluence, pulse duration, and the presence of water-cooling, critically influences its effects on the tooth structure [43]. Previously Nalla et al. observed that minor flaws in dentine (approximately 250 mm) do not significantly affect mechanical integrity under simulated clinical conditions [44].

Furthermore, no substantial differences were visualised on the SEM images between the laser groups. This is in accordance with a previous study that showed no significant morphological changes in dentine treated with Er:YAG or Nd:YAG lasers parallel to the surface [45].

In the present study, the fracture strength of dentinal beams subjected to different laser treatments (Er:YAG, Er,Cr:YSGG, and 940 nm diode) was assessed using a four-point bending test. The results from cyclic loading of the specimens showed that various laser treatments did not cause a reduction in fatigue resistance compared to control (Figure 4). It is to be highlighted that the different laser delivery methods may be a key factor in our study and may contribute to the observed differences or similarities in the results.

Arola and Rouland investigated the fatigue crack growth rate in relation to dentine tubule density and orientation. They concluded that the tubule orientation significantly impacts crack propagation. Accordingly, in this study, all irradiated dentine surfaces were marked to ensure consistent orientation during mechanical testing. Moreover, during the bending test, the samples were positioned such that the laser irradiated surface was in the tension side [46]. This testing configuration applies tensile stress to the irradiated dentine surface between the central supports, effectively promoting crack initiation and propagation from laser-induced flaws. The relatively broad tensile stress field increases the probability of engaging surface and subsurface defects, thereby enhancing the sensitivity of the method to detect treatment-induced damage or microstructural alterations.

The efficacy of laser-assisted endodontics remains an ongoing debate. Despite numerous studies highlighting the advantages of the use of laser in nonsurgical endodontic procedures since the 1970s, widespread clinical adoption has been hindered by concerns over potential thermal damage [7, 47]. Erbium lasers have demonstrated the ability to remove the smear layer from dentinal tubules [1], a key objective in root canal decontamination. The influence of erbium lasers on dentine has been extensively studied [12, 39, 48–50], underscoring the importance of selecting appropriate parameters to prevent adverse mechanical effects. Since tooth fractures result from multifactorial influences, laser settings can substantially impact dentine properties. Various pulse durations, repetition rates, energy, and power levels have been analysed in relation to micromorphological changes, ablation depth and efficiency [12, 39, 48–50].

Erbium lasers have FDA approval for cleaning, shaping, and enlarging the root canal system. Earlier versions of endodontic laser tips emitted energy in a straight line (bare-ended), whereas modern conical radial-firing tips provide lateral emission in a 360-degree pattern. A key limitation of bare-ended tips is their potential to cause irrigant extrusion into apical tissues. Conversely, three-dimensional activation allows deeper penetration into complex canal networks without excessive apical extrusion [7, 51]. A previous study by Hmud et al. [52] investigated the ability of the 940 and 980 nm diode lasers to generate bubbles within aqueous solutions. However, a common misconception in the research community is that all bubble formations in water equate to cavitation. Unlike erbium-laser-induced cavitation, where bubble formation and collapse occur within microseconds, diode-induced bubbles form more slowly and persist longer.

Additionally, diode lasers operate at low power with longer pulse durations (milliseconds), lacking the shock wave characteristics of pulsed erbium lasers. The collapse of erbium-induced cavitation bubbles generates a mechanical pressure front, which can be captured using high-speed imaging [7, 49]. This study aimed to evaluate the potential structural changes in dentine that might influence fatigue strength after laser-assisted endodontic treatment. The experimental design compared intracanal irradiation using a 940 nm diode laser in dry mode to LAI in root canals with Er:YAG (2,940 nm) and Er,Cr:YSGG (2,780 nm). The optical properties of each wavelength are crucial in determining its applications and limitations. Mid-infrared erbium wavelengths are highly absorbed by water and hydroxyapatite, making them ideal for dental hard tissue removal. In contrast, the 940 nm diode exhibits strong absorption in melanin and haemoglobin, optimizing bacterial decontamination.

This study employed pulse durations clinically relevant for endodontic procedures. With erbium lasers, water is typically used for root canal cleaning. In the presented research, saline solution was the sole irrigant to isolate the effects of laser irradiation. Vapour bubble formation within the irrigant generates cavitation effects. The primary vapour bubble expands during the laser pulse before collapsing, producing shock waves (travelling at supersonic speeds) and acoustic waves (travelling at sonic speeds). The implosion of the primary bubble generates secondary cavitation bubbles, creating shear forces along the root canal walls, which enhance cleaning efficacy. However, the precise pressure effects of these shock waves remain incompletely characterised [19, 29, 52].

Peeters and De Moor demonstrated that the pressure variations within the root canal using Er,Cr:YSGG with conventional LAI (laser-activated irrigation) technique at 0.75W were significantly lower than 1.75W regardless of fibre tip type and irrigation solutions used (NaOCl or EDTA). Moreover, the pressure increased as the laser tip approached the apical region [53]. Among all tested protocols, Er:YAG using a PIPS fibre tip exhibited the least periapical extrusion [54]. Unlike PIPS tips (Er:YAG), the RFT tips (Er,Cr:YSGG) can be used for bacterial decontamination in dry mode, like the 940 nm diode, which is commonly utilised for deep root canal disinfection. Previous studies could present open tubules for erbium lasers due to the cavitation effect. In this study, the results (SEM images) showed greater opened tubules compared to the control group. However, these findings were not as excessive as in previous results [55], because of the testing setup.

Hibst und Keller [56] stated that adjusting the water spray could minimise tissue dehydration and reported that free-running erbium laser shock waves did not cause destructive effects, contrasting the findings of Staninec et al. [43], who observed damage with ultra-short pulses using a q-switch laser. Conversely, ultrashort pulses in the latter study were applied dry.

This study had some limitations. One limitation was that the space is not exactly as in narrow root space. Also in real clinical situation, the emitted laser energy will be transmitted through the dentine, which is not the case in this test set-up mould. Due to cavitation effect, some of the energy is lost over the edges due to possible tiny movements in beams, even though the beams were stabilised with hand-held instrument during irradiation. The power emitted will be diminished on the proximal end of the fibre tip differing from the parameters shown on laser display because of the calibration factor [7, 45]. This test setup was extremely standardised as all groups underwent procedures in the same root canal mould, contrasting real clinical situations with alternating canal length, shape, and diameter. The laser beam is directed via RFT, having lateral emission, towards the canal surface. In the real clinical situation, however, the probable wall contact cannot be constantly avoided. This limits the optimal positioning of the fibre tip and the consequent fluid movement inside the root canal. Therefore, the Er;Cr:YSGG and 940 nm diode can undertake much more wall contact. Another inconsistency with these protocols is the manual helical movement of the fibre tip. In contrast, the PIPS tip is kept still in the pulp chamber and, therefore, independent of intracanal tip positioning. One drawback could be the artificial nature of the used mould. The non-biological wall material has a smooth surface and an exclusively formed shape with a straight canal compared to the natural root canal anatomy. Also, saline solution or distilled water (Er,Cr:YSGG) was used as the only irrigant instead of NaOCl or EDTA. This was done, since the goal of the study series was to focus solely on the mechanical effects of the laser irradiation, thus excluding all other commonly used irrigants [48].

Several studies have examined resin-dentine bonds focusing on hydrolytic degradation [57] or biofilm-acid-induced deterioration [58, 59]. Mutluay et al. reported that biofilm-induced degradation significantly reduces dentine strength and fatigue resistance. The acids produced by biofilm lead to extensive dentinal surface demineralisation [60]. By leveraging photoacoustic effects, erbium lasers effectively eliminate biofilm, potentially preventing acid-induced degradation. This may also benefit the long-term durability of the tooth overall. Future research could include commonly used irrigants and adding dentine debris, smear layer, or biofilm. The natural biofilm differs in composition and structure from the smear layer, which could have a dissimilar outcome in fatigue behaviour when exposed to laser irradiation [60].

## Conclusion

Based on the findings of this study, it can be concluded that the 940 nm diode laser, as well as Er:YAG and Er,Cr:YSGG lasers at the tested settings, did not compromise the fatigue strength of root dentine. Therefore, these lasers may serve as a valuable adjunct to existing protocols for root canal cleaning and disinfection.

## Data Availability

Data will be made available on request.
